# Gender bias in special issues: evidence from a bibliometric analysis

**DOI:** 10.1007/s11192-023-04639-z

**Published:** 2023-02-20

**Authors:** Magdalena Formanowicz, Marta Witkowska, Weronika Hryniszak, Zuzanna Jakubik, Aleksandra Cisłak

**Affiliations:** Center for Research on Social Relations, SWPS University of Social Sciences and Humanities in Warsaw, Warsaw, Poland

**Keywords:** Gender bias, Bibliometric analysis, Special issues

## Abstract

Even though the majority of psychologists are women, they are outnumbered by men in senior academic ranks. One reason for this representation bias in academia is that men favor other men in decision-making, especially when the stakes are high. We tested the possibility of such bias in a bibliometric analysis, in which we coded editors' and authors' gender in regular and special issues, the latter considered of higher scientific prominence. We examined all special issues from five prominent scientific outlets in the fields of personality and social psychology published in the twenty-first century. Altogether, we analyzed 1911 articles nested in 93 sets comprising a special issue and a neighboring regular issue treated as a control condition. For articles published in special (but not regular) issues, when there were more men editors, more men first-authored and co-authored the work. This pattern suggests how gender bias can be perpetuated within academia and calls for revising the editorial policies of leading psychology journals.

Men outnumbering women in high-status academic positions is a well-documented fact. It is evident even in the domains like psychology, where in the US context, women constitute the majority of students in undergraduate and graduate studies (71% and 78% respectively; National Science Foundation) and of members in the American Psychological Association (58%; APA, 2017) and the Society for Personality and Social Psychology (51%; SPSP, 2016). Notably, the proportion of women among associate and full professors in social and personality psychology is 45.8% and 28.1%, respectively (Johnson et al., 2017). We label this higher presence of men versus women in high-status academic positions irrespective of base rates a representation bias.

One explanation for this representation bias comes from the social role theory, according to which men and women are expected to possess attributes that equip them for sex-typical roles (Eagly, 1987). Stereotypically masculine, but not feminine, traits are related to agency and therefore substantially overlap with traits associated with high-status scholarship (Leslie et al., 2015; Miller et al., 2018). Accordingly, the decision-makers may favor men in a selection process, as they may match the stereotype more than women, according to the role congruity principle (Eagly & Karau, 2002).

However, in line with social identity theory and the ingroup favoritism phenomenon, the general tendency to prefer men over women for high-status positions can depend on the decision-makers’ gender (Tajfel, 1982). Specifically, representation bias can be more evident in a predominantly masculine environment, as men benefit more from this cultural arrangement. In this case, the lower presence of women in high-status positions is particularly eminent when men make selection decisions that can favor their ingroup, that is other men. If more women were among the decisive bodies, the asymmetry could be diminished, as women do not share the male ingroup bias with men.

The current research examines gender representation in publication outlets of various academic prestige. Specifically, we compare gender representation within authorship in both the special and regular issues of five personality and social psychological journals. We focus on differences pertaining to the authorship because the editorial roles are always considered as prestigious for both regular and special issues alike. In terms of publishing, however, special issues in comparison to regular issues in the same journals, are prominent and sought-after outlets in science for authors, as they indicate the author’s expertise on a given topic and contribute to that expertise through higher citation rates (Conlon et al., 2006). Under the assumptions of social role theory, there should be an overall higher presence of men than women in such high-status outlets. However, if some sort of ingroup bias is additionally at play, the number of women as authors could vary depending on the gender composition of the editorial team. Specifically, we would observe that a higher number of men in editorial teams would lead to a higher representation of men as authors. For regular issues that bear less association with high status (Conlon et al., 2006), the gender composition of editorial teams should be less related to the authors’ gender composition.

We consider this investigation important because representation bias can be a crucial factor in maintaining gender bias within academia. Positions of power shape collective associations regarding who can be influential in science, and the power of these stereotypes also affects discrimination with respect to hiring, advancing, granting research funding, or publishing. Representation bias not only contributes to the equality of opportunities for women and men but also eventually affects the quality of science. Diversity in the academic pool contributes to bringing diverse experiences and perspectives, thus fueling scientific innovation and progress (Formanowicz, 2021; Hofstra et al., 2020). Therefore, analyzing which factors may contribute to the persistence of representation bias can address both the issue of equality and the quality of scientific discovery. Below, we review in more detail the evidence for representation bias in academia, stereotypical beliefs that associate men with high-status scholarship, and findings and theorizing that suggest that the preference for selecting men for high-status positions can be particularly strong among men.

## Representation bias in academia

As we already mentioned, one of the most obvious manifestations of representation bias in academia pertains to the employment hierarchy. The higher the status of the position, the lower women’s presence becomes (Gruber et al., 2021). For example, female and male PhD graduates from prestigious universities find good employment at different rates, with men finding better placements in jobs than women (Clauset et al., 2015). Additionally, research has captured gender asymmetry in promotions (i.e., men advance faster than women; Ginther & Kahn, 2006, 2015) and deanship representation (i.e., overrepresentation of men among deans; Bilen-Green et al., 2008). Importantly, while longitudinal data indicate that the gender gap in promotions and the tenure rate is shrinking (Webber & Canché, 2015), gender disparities at higher levels of academic hierarchy still exist (Carter et al., 2017; Ginther & Kahn, 2015), further exacerbated by the increasing precarity of lower level academic positions that disproportionately affects women in comparison to men (Minello, 2020).

Differences in the treatment of men and women in high-status positions also extend to payroll (APA, 2017; Ceci et al., 2014). When hired, women in academia are paid less than men, even though they demonstrate similar scientific productivity (Brower & James, 2020; Frandsen et al., 2020). The difference in salary is especially pronounced among associate and full professors (Gruber et al., 2021). Finally, gender bias in academia is also present in reference to other financial indices, such as grant success rates (Bornmann et al., 2007; van der Lee & Ellemers, 2015), even at the very early stages of an academic carrier (Wijnen et al., 2021).

Representation bias, however, can also manifest more subtly in terms of access to professional prestige, which can determine whose voice is heard loud and clear and who has more influence in shaping the academic field. Here, men are more often invited as speakers at conferences and colloquia (Nittrouer et al., 2018) or to prepare editorial introductions that may shape their respective fields, such as health or medical sciences (Chang & Cesare, 2020). Men are also likelier to be awarded and honored by others for their contributions to the field (Diener et al., 2014).

The same pattern can be detected in bibliometric disparities. While women are less likely to be found in prestigious first and last author positions across fields (Odic & Wojcik, 2020; West et al., 2013), recent findings have also shown that middle authorship, which is important for career development as well, is susceptible to the gender gap (Fleischmann & Van Berkel, 2021). An analysis by Odic & Wojcik (2020) also shows that articles written by men are cited roughly 1.3 times more often than those by female authors. This tendency persists even when women publish in top-tier review journals. Female first-authored papers are also less likely to be included in graduate-level syllabi, which can be explained neither by the relative availability of male first-authored papers in the published literature nor by an alleged preference for including classic rather than recent papers (Skitka et al., 2020). Men are also overrepresented among reviewers playing a crucial role in the gatekeeping of scientific publishing (Zhang et al., 2022). Overall, disparities in employment and scientific recognition indicate that men and their work are overrepresented in high-status roles, which leads to the enhanced visibility and recognition of male members of academia.

## Factors contributing to gender representation bias

One reason for the higher appreciation of men than women academics in high-status positions may arise from the overlap of cultural stereotypes of scientists and men and the resulting association of science (and scholarship) with masculinity (Eagly & Miller, 2016; Leslie et al., 2015; Miller et al., 2015, 2018). This is particularly evident in field-specific ability beliefs that address a common perspective that some fields require natural-born talent or brilliance (Leslie et al., 2015). Accordingly, adults and children implicitly associate high levels of intellectual ability (e.g., brilliance, genius) with men more than with women (Storage et al., 2020), and it has been found that women are underrepresented in disciplines that are associated with genius (Cimpian & Leslie, 2017; Meyer et al., 2015).

The male-brilliance stereotype operates within academia through at least two different routes. First, it is related to a selection bias favoring candidates who match the stereotype. For example, letters of recommendation for college (LaCroix, 1985) and graduate schools (Watson, 1987), when conveying stereotypically masculine traits (labeled also as agentic and pertaining to efficiency or goal achievement) for men and stereotypically feminine traits (labeled also as communal and pertaining to warmth or kindness) for women, clearly indicate who is more matched with the stereotype of a scholar. Accordingly, those described with agentic traits are hired more often than those described with communal traits (Madera et al., 2009). In a similar vein, male applicants chosen for medical school were described with adjectives that highlighted their brilliance and praised with research-related adjectives, even though objective criteria showed no differences in qualifications with their female counterparts, for whom communal and teaching abilities were referenced (Trix & Psenka, 2003). The power of the cultural association between masculinity and high-status scholarship can be inferred from the fact that not only men but also women engage in a selection bias that favors men in academia (Moss-Racusin et al., 2012). This is particularly evident in the so-called queen bee effect, which is a phenomenon observed within male-dominated domains where women who attain high-status positions engage in sustaining gender inequalities themselves, specifically if the system supports male dominance and discrimination against women (Derks et al., 2011; Ellemers et al., 2004; Faniko et al., 2020).

The second route through which the male-brilliance stereotype might operate is self-selection bias. Women can avoid fields that require brilliance, given that they perceive themselves as not fitting into that role. Furthermore, women share the explicit and implicit association of brilliance, a frequent attribute of a scientist, with men—they associate brilliance with men more than with their own gender group (Storage et al., 2020). These associations have profound consequences, as both girls and women apply them when shaping their career choices (Bian et al., 2017, 2018). In reverse, men could internalize the brilliance stereotype. This is evident for example in how male and female scientists present the importance of their own work, with men using terms like “excellent” or “unique” more often, even though their research itself may not in fact be more novel or innovative (Lerchenmueller et al., 2019). This bias in self-praise may explain why female-authored grant proposals may receive lower evaluations, even from blind reviewers (Kolev et al., 2019). To sum up, role stereotypes permeate general norms and preferences, thus they can contribute to sustaining representation bias through the higher preference for men candidates, along with the lower willingness of women to apply to these fields.

## Male ingroup bias as a factor exacerbating gender representation bias

Both the biased selection and self-selection phenomena speak to the pervasiveness of the cultural stereotype conflating high-status scholarship with masculinity. However, an additional motivational factor might also affect representation bias: male ingroup bias.

Favoring one’s own ingroup is a well-known phenomenon in psychology. It refers to people’s tendency to prefer groups associated with themselves as confirmation of their high self-esteem (Allport, 1954; Tajfel et al., 1971). Such ingroup love does not necessarily equal outgroup hate (Allport, 1954, Golec de Zavala et al., 2013), but it can lead to outgroup derogation, especially under conditions of competition over material resources or power (Allport, 1954; Brewer, 1999). Accordingly, studies show that ingroup bias is more likely to occur under conditions of limited resources or scarcity, in which it is hard to serve both ingroup and outgroup members in a way that resources are evenly distributed (Chae et al., 2022). We consider this phenomenon worth examining in the context of gender in academia, which (beyond cooperation) is a field of competition over prestige, power, and limited resources such as prestigious publications in special issues which are less frequent than publications in regular issues.

Given the stakes, high-status group members are particularly prone to exhibit ingroup bias (Scheepers, 2009, 2017) because they are motivated to maintain their privileged position. Accordingly, the magnitude of the representation bias in academia may depend on the gender of the decision-makers. As male scientists benefit more from this cultural arrangement, they can be more susceptible to representation bias, as it directly benefits their ingroup. Therefore, men-dominated environments could favor other men, as this contributes to maintaining men’s privileged position. In contrast, where there are more women in the decision-making body, the male ingroup bias can be less prevalent, thus diminishing representation bias.

Indeed, research has indicated that, in high-status male-dominated prestigious research universities, men are favored in the selection process (Sheltzer & Smith, 2014). Furthermore, at high-status major psychology conferences, which are prominent outlets for one’s work and landmarks of scientific visibility (Johnson et al., 2017), there are, in general, fewer women speakers. The representation of women in the symposia, however, has been found to vary with the presence versus absence of women in the symposium chair. When men are in charge of the symposium, men are predominantly invited to present. A similar pattern has been observed for disciplines other than psychology (Isbell et al., 2012). Finally, when women have the first author position in an article, gender balance among authors is higher (though still not present) in comparison to when men hold this prominent authorship position (Jemielniak et al., 2022). Overall, there is scattered but consistent evidence, that when men make the decision there are fewer women involved across a range of academic endeavors. 

## Overview of the current research

Career development is driven by publishing in top journals, which serve as a system for the collection and dissemination of scientific knowledge. Some of those journals, along with editorial materials (Chang & Cesare, 2020), also run *special issues*, which are collections of papers on specific topics that provide insight into the most significant research and findings on a given subject. These issues find popularity and prestige, as they have higher citation rates than regular issue articles (Conlon et al., 2006) and provide considerable influence on the bibliometric profile of the journal by attracting more immediate citations and more overall citations (Smith, 2012). Thus, whoever is invited to write articles for special issues obtains the opportunity, not only to pursue a personal career but also to influence the trajectory of the field.

Given the high status of special issues among publication outlets, in this article, we examine the representation of women as authors in special issues (vs. regular issues) in relation to the editors’ gender. This investigation follows recent anecdotal evidence (Ledgerwood et al., 2015): in two of the special issues in *Perspectives on Psychological Science* focused on the future of psychology following the replicability crisis, the editors were all men, as were the majority of the authors. We extend this anecdotal finding to a systematic analysis in which we examined whether, in special issues, a higher number of men on editorial teams leads to a higher representation of men as authors. For regular issues, which bear less association with high status, the gender composition of editorial teams should be less related to the gender composition of articles’ authors. On top of the analysis examining the potential male ingroup bias, we also investigate the gender composition of the editorial teams of regular and special issues, a topic that, in our opinion, has not received proper attention in the field of social psychology. Given, however, that being on the editorial team is a prestigious appointment in special and regular issues alike, in line with the previous argumentation, we expect women to be less represented than men regardless of the outlet.

## Methods

### Sample of articles

We identified five prominent social psychology journals that regularly publish special issues: *European Journal of Social Psychology* (*EJSP*), *British Journal of Social Psychology* (*BJSP*), *Group Processes and Intergroup Relations* (*GPIR*), *Journal of Experimental Social Psychology* (*JESP*), and *Political Psychology* (*PP*). Within each outlet, we identified all special issues published between 2000 and 2020. For each special issue, we created a set comprising the special issue itself and one regular issue that preceded the special issue. If a special issue was preceded by another special issue or an issue that was already included in the dataset, we took the nearest regular issue available following the respective special issue. Therefore, each set comprised one special issue and one regular issue. We also classified special sections, symposia, and millennia as special issues provided, they were edited by guest editors. We did not include such sections if they were not edited by a guest editor or if there was no editor information. If within a volume there was a special section and regular articles, the latter were added to the control group. We also decided not to include virtual special issues because they are usually thematic collections composed of articles published in previous years (in regular issues), and thus, they are not articles written and submitted to be included in special issues. From the sample of 93 sets, we deleted 94 editorials, 82 book reviews, 13 corrigenda or errata, two dialogues (as they comprised multi-article entries), eight retractions, eight ​​presidential addresses, six award addresses, one *in memoriam* note, six millenium articles as we could not track down the editors of the serie, six "briefly noted" entires and one thematic review essay. The final sample consisted of 93 sets comprising 1911 articles, of which 764 were special issue articles, and 1147 were regular articles (regular issues were coded as 0 and special issues were coded as 1). For the list of all articles, including the exclusions, see materials available at https://osf.io/2ykwm/?view_only=f2a64e3a714a46a9a0272a832a4a433e. For a summary of the journal and sample statistics, see Table 1.

**Table 1:** Descriptive statistics regarding the collected sample of articles, considered for each journal separately, calculated at the set level

Journal	IF_2021_	Sets *N*	Issue type	Articles *N*	Percentage of female editors *M* (*SD*)	Percentage of female authors *M* (*SD*)	Gender of the first author *M* (*SD*)
*BJSP*	4.691	9	Regular	151	50.00 (0.00)	45.42 (7.61)	0.51 (0.15)
			Special	65	45.37 (32.57)	34.95 (18.16)	0.39 (0.24)
*EJSP*	3.376	19	Regular	239	49.12 (32.14)	42.68 (9.11)	0.44 (0.14)
			Special	199	42.28 (35.61)	51.30 (14.95)	0.56 (0.18)
*GPIR*	3.129	33	Regular	262	0.00 (0.00)	46.91 (13.58)	0.51 (0.20)
			Special	259	30.05 (31.86)	46.17 (16.38)	0.50 (0.24)
*JESP*	3.603	7	Regular	280	0.00 (0.00)	41.50 (6.45)	0.45 (0.10)
			Special	74	35.71 (39.00)	46.55 (18.07)	0.55 (0.31)
*PP*	4.333	25	Regular	215	38.96 (31.37)	33.92 (14.83)	0.33 (0.19)
			Special	167	49.33 (43.43)	35.37 (19.25)	0.32 (0.23)

### Authorships and editors-in-chief

Each article was coded with respect to the number of authors and their gender. Gender was established by a web search of departmental or private webpages, photographs, or other online records (such as genderdize.io). In four cases, the gender could not be determined, or the authors classified themselves as non-binary. Given the gendered hypothesis of this article, on those occasions, we coded the author’s gender as a missing value and reduced the number of authors for the respective article. Editors for the regular issues were determined from the journal webpage or by communicating with each journal’s managerial office; for special issues/sections, we coded the authors of the editorial as editors. Thirty percent of the coding for authors’ and first authors’ gender were repeated by independent raters. In both cases, the inter-rater agreement was high (*κ* > 0.90, *p* < 0.001).

## Results

### Preliminary analyses

As our first step, we compared the percentage of female editors in special (*M* = 39.64, *SD* = 36.73) and regular issues (*M* = 25.35, *SD* = 31.06), and these values were significantly different *t*(179.04) = 2.87, *p* = 0.005, *d* = 0.42 (equal variances not assumed). It should be also noted that, in both types of publication outlets, the percentage of female editors was significantly lower than 50% indicating gender balance *t*(185) = − 6.89, *p* < 0.001, *d* = 0.51 and than 60% that is the actual percentage of women in the psychological field (APA, 2017) *t*(185) = − 10.82, *p* < 0.001, *d* = 0.79.

In our second step, we compared the percentage of female authors in special (*M* = 43.26, *SD* = 18.02) and regular issues (*M* = 42.01, *SD* = 13.16), which were not significantly different *t*(168.41) = 0.54, *p* = 0.589, *d* = 0.08 (equal variances not assumed). Also the distribution of male and female first authors was similar in special (*M* = 0.46, *SD* = 0.24) and regular issues (*M* = 0.45, *SD* = 0.19) *t*(172.34) = 0.45, *p* = 0.650, *d* = 0.07 (equal variances not assumed). Again, it should be noted that, in both types of publication outlets, the percentage of female authors was significantly lower than 50% indicating gender balance *t*(185) = − 6.38, *p* < 0.001, *d* = 0.47) and than 60% that is the actual percentage of women in the psychological field *t*(185) = − 15.04, *p* < 0.001, *d* = 1.10. In addition, the distribution of females and males as the first authors was unequal in favor of men *t*(185) = − 3.02, *p*  =0.003, *d* = 0.22. The mean for coding of the first author’s gender was also significantly lower than 0.60, referencing the actual ratio of women in the psychological field *t*(185) = − 9.30, *p* < 0.001, *d* = 0.68.

Finally, we observed the following relationship between the collected variables. The percentage of female editors was not related to the percentage of female authors *r*(184) = 0.10, *p* = 0.19. The frequency of female first authorship was not related to the percentage of female editors *r*(184) = 0.12, *p* = 0.10, but it was positively correlated to the percentage of female authors* r*(184) = 0.83, *p* < 0.001.

### Hypotheses testing

To examine the main hypothesis of this research (i.e., that the number of women authors varies in relationship to the percentage of female editors in special but not in regular issues), we employed a regression model using MPlus7 (Muthén & Muthén, 2019). We used two separate dependent variables: the percentage of female authors and the gender of the first author.

**Fig. 1:**
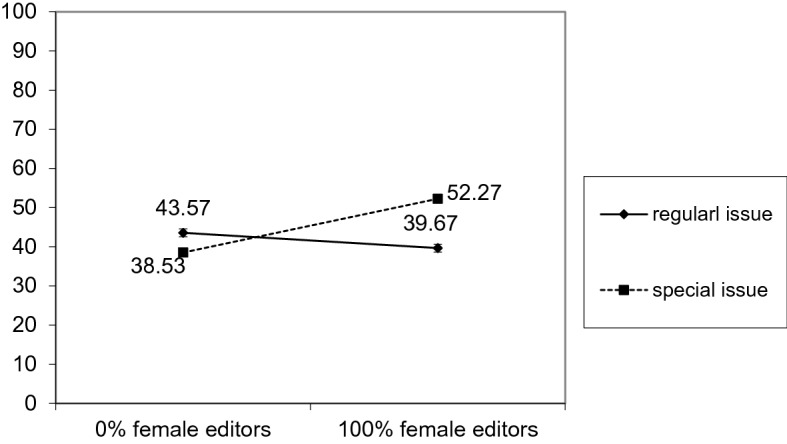
The interaction effect of high vs. low percentage of female editors and publication status (special/regular issue) on the percentage of female authors

First, we examined the percentage of female authors of articles as a dependent variable and as predictors of the type of issue (special vs. regular), the percentage of female editors of the issue (mean-centered), and their interaction. Because cases were non-independent in this model, we nested articles in sets to obtain a robust standard error (SE) estimation. As predicted, we found a significant interaction effect *B* = 0.17; *SE* = 0.06; *p* = 0.004; illustrated in Fig. 1. A simple slope analysis revealed that the percentage of female authors for special issues increased proportionally to the percentage of female editors *B* = 0.14; *SE* = 0.05;* p* = 0.007. The simple slope for regular issues was not significant *B* = − 0.04; *SE* = 0.03; *p* = 0.249; that is, we were unable to detect any relationship between the percentage of female authors and female editors in regular issues. When the nonsignificant parameters (i.e., the effects of the percentage of female editors and the type of issue) were constrained to 0, the final model fitted the data very well *RMSEA* = 0.00, 95% *CI* [0.00; 0.04]; *CFI* = 1.00; *SRMR* = 0.01, *χ*^*2*^(2) = 1.05, *p* = 0.593.

**Fig. 2:**
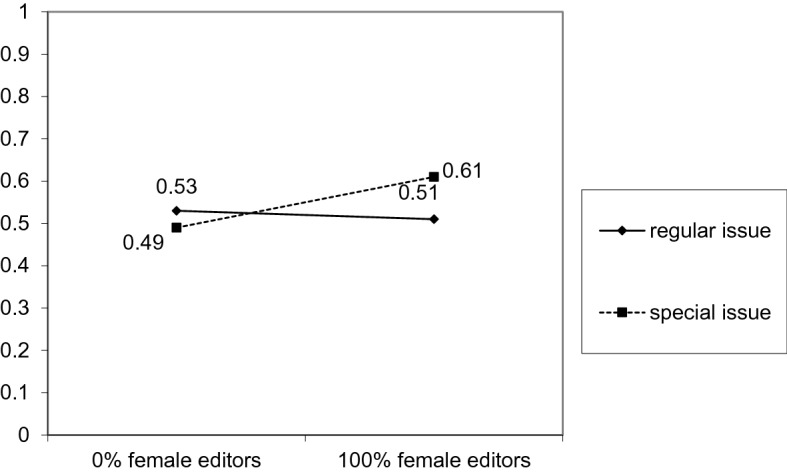
The interaction effect of high vs. low percentage of female editors and publication status (special/regular issue) on the probability of females being first authors

Second, we repeated this analysis for the gender of the first author as the dependent variable (coded 0—man, 1—woman) and found a significant interaction effect *B* = 0.01; *SE* = 0.002; *p* = 0.003. A simple effects analysis revealed that the likelihood of females becoming first authors for special issues was positively related to the percentage of female editors *B* = 0.01; *SE* = 0.002; *p* = 0.005. The effect for regular issues was not significant *B* = − 0.001; *SE* = 0.001; *p* = 0.318; that is, we were not able to detect any relation between the frequency of females being first authors and female editors in regular issues. When the nonsignificant parameters (i.e., the effects of the percentage of female editors and the type of issue) were constrained to 0, the final model fitted the data very well *RMSEA* = 0.00, 95% *CI* [0.00; 0.04]; *CFI* = 1.00; *SRMR* = 0.02, *χ*^*2*^(2) = 1.00, *p* = 0.61. (See Fig. 2).

## General discussion

In this research, we investigated the presence of a gender ingroup bias in special and regular issues of personality and social psychology journals. We examined all the special issues published by five prominent journals in the twenty-first century and compared them to the adjacent regular issues of those journals. First, we found evidence of gender bias in terms of the editorial teams, the overall gender composition of publications’ authors, and the proportion of female first authors. The two latter results are congruent with previous research (Odic & Wojcik, 2020). However, we are not aware of any other research documenting the low representation of women in editorial teams. This pattern is in line with the role congruity principle, as these results document a lower presence of women in the masculine domain of the prestigious editorial role. Interestingly, in regular issues we observed a significantly lower number of women as editors than in special issues. This can reflect the fact that a regular editorial position allows to shape policy of a journal for many years rather than for one (special) issue only, and in this way is of the highest status in the publishing hierarchy. Nevertheless, in both types of publication outlets, women were not fairly represented in reference to their presence in the field of psychology.

Second, we also found that the percentage of women as authors as well as the likelihood of being a female in the first author position in regular issues was not moderated by the gender composition of the editorial teams. In the case of special issues, however, when more male editors were on the team, there was a smaller percentage of women as authors as well as smaller likelihood of a woman being in the first author position. This finding is in line with the ingroup bias hypothesis and sheds new light on psychological processes underlying editorial practices.

At first sight, these results may seem to document gender symmetry. Figure 1 shows a linear relationship in which predominantly male editorial teams publish papers of predominantly male authors, whereas predominantly female editorial teams publish papers of predominantly female authors. However, the graph presents a model estimation. In reality, the average number of women on editorial teams for special issues was low. More importantly, even teams comprising 100% women (*N* = 17) accepted articles with an average of 50.35% female authors, which is at the level of gender parity and still below the actual representation of women in psychology. Should the average proportion of female editors in the special issues be, however, higher, a higher proportion of female authors that is closer to the proportion of women in the field may also be expected. Thus, the findings presented here may also suggest the potential means for closing the gender gap by increasing the number of female editors. The fact that we did not observe ingroup favoritism among women editors deserves further research, however, it is in line with previously studied phenomenon of justifying inequalities (Jost et al., 2003) by disadvantaged groups related to internalization of stereotypes.

We interpret the observed pattern of results as reflecting ingroup bias that comes into play when a valued goal is at stake—in this case submission to a high-status outlet. However, the observed pattern of results can also be explained by alternative ingroup motivations. For example, men’s and women’s professional networks largely differ due to their different career experiences (Atchison, 2018). Specifically, men may know more men, and thus, their professional lives happen in a predominantly masculine environment. Accordingly, in contemporary academia, men overall collaborate and publish more with other men (Araújo et al., 2017, Abramo et al. 2019). Furthermore, social networks of high-status groups and men alike show similar patterns of spreading further than networks of low-status groups and women, whose networks are denser and more localized (Smith et al., 2020). Therefore, when putting together a special issue, men can reach out to other men, as well as to men from diverse backgrounds, due to their availability in their networks. Such practices at the level of an individual editor may simply be a matter of ease in finding collaborators not motivated by the ingroup preference. However, accumulated across many instances, such practices can create a pattern of bias at the institutional and editorial policy level, and not correcting for such tendencies could produce gender disparities.

The findings presented here are important for the following reasons. Special issues are aimed at summarizing the main achievements in the field, opening new research avenues, and setting the standards in science. Indeed, special issues attract broader readership, what is reflected in their higher citation rates, both in the short and long term (Smith, 2012). However, selecting authors for special issues and the dominance of single authorship can lead to a smaller diversity among authors (Das, 2017). This can work to the disadvantage of scientific excellence because diverse teams tend to produce better outcomes (Skilton, 2008). Caring for an adequate representation of women in the author pool can contribute to the inclusion of different voices and perspectives, which, in turn, affects the innovation rate and the quality of insight (Hofstra et al., 2020).

This finding also has societal implications. Although it presents a specific manifestation of gender bias in academia, in a broader sense, it adds another position to the catalog of instances of gender bias that have been revealed and, therefore, can be effectively counteracted. Documenting each instance of gender bias counts (Formanowicz, 2021), as it allows for broadening diversity awareness and, thus, taking effective countermeasures. Importantly, in line with social perception studies, the awareness of implicit attitudes brings an obligation to foresee the discriminatory results that, in turn, stand behind the perceived moral responsibility (Redford & Ratliff, 2016). Thus, social and personality psychologists as a community may be perceived by society as especially obliged to foresee such effects and thus hold the responsibility for them. In line with the findings presented here, including more women in the editorial teams of special issues, may in turn increase the number of female authors and visibility of their work in the field, thereby reinforcing gender equality efforts in academia.

### Limitations and future directions

One of the main limitations of this research is the correlational nature of the presented data, which precludes causal inferences. Our analysis covered the authorship of the articles published by the journals, as we did not have access to the broader sets of manuscripts out of which these publications were selected. It is thus equally likely that the pattern we observed is the result of gatekeeping by male editors, self-selection of female authors, or both.

Specifically, the pattern observed in the current dataset may be due to different editorial policies and different patterns of self-selection in special issues compared to regular issues. First, as special editorial teams usually emerge ad hoc and operate as teams for shorter terms, they objectively have fewer opportunities to discuss and revise their approaches than regular teams. As a result, those teams may both get into contact and attract more authors who are similar to the editors themselves. Thus, gender networking may, by default, affect to a higher extent the final selection of articles accepted into special compared to regular issues. Another potential alternative explanation pertains to the fact that special issues are centered around a specific topic. As women and men can be involved differently in some topics more than others (Kim et al., 2022), special issues can reflect those potential differences and their authorship indicates who works on a specific topic rather than a bias of some sort. While this explanation seems plausible, we employed in our analysis journals of similar profiles in social psychology, which generally cover topics of interest to both women and men scholars. Furthermore, in the data, we do not observe evidence for women editors choosing predominantly women as authors—as mentioned above. All female editorial teams (*N* = 17) accepted papers with on average equal number of women than men as authors (*M* = 50.35%). In all male editorial teams (*N* = 34), however, the percentage of women as authors was pronouncedly lower (*M* = 37.07%). The striking discrepancies in both the number of special issues edited by all men and all women editorial teams as well as in representation of women as authors depending on the gender of the editors suggests some sort of ingroup bias in case of men only. Whether this bias is driven by status of special issues remains only one valid hypothesis. Future research should investigate alternative potential explanations.

Furthermore, future research should also examine the generalizability of the findings obtained in psychology to other disciplines to see whether the pattern of the results varies as a function of discipline and the situation of women in the respective field. There are two possible pathways here. On the one hand, the result obtained in this research is representative to psychology or more generally the fields of life, social and behavioral (LSB) sciences but not to the natural sciences, technology, and economics (NTE). These two areas substantially differ in gender representation (van Veelen & Derks, 2022). For NTE, the percentage of women as bachelor students and assistant professors is on par, while in LSE a large proportion of female students significantly drops at the level of assistant and full professorship, which is considered a signal of a thicker glass ceiling effect in LSE in comparison to NTE. As a result, patterns obtained in the current work may not hold within the NTE domain—a hypothesis that awaits future research efforts, as the main focus of this work is placed on psychology. On the other hand, similar results could be expected for other disciplines as well, given that across scientific disciplines, the scholarship is associated with masculinity and status (Van Veelen & Derks, 2021). In NTE disciplines a glass ceiling effect exists as well albeit smaller than in LSE. Accordingly, bibliometric analyses have noted that also outside of social sciences women are less represented in high status outlets, for example they are less invited to writing influential editorial pieces (Chang & Cesare, 2020). Similarly, an analysis of changes in the pattern of proportion of women authors during the COVID-19 pandemic shows a complex picture rather than a simple LSE and NTE division (Jemielniak et al., 2022). The highest decrease in the number of women authors (during the pandemic in comparison to previous years) was observed in disciplines as different as psychology, mathematics, and philosophy. This bias however, was not evident for social science in general as well as not for many life sciences. The complex picture observed in the current research precludes us from making any generalizable conclusions, leaving it for future research.

While in this research we focused on the binary gender and the visibility of research of those academics who identify as women versus men, similar effects may be potentially observed in the case of other disadvantaged (compared to advantaged) groups including categories such as race, ethnicity, or sexuality. With the increasing numbers of persons who reveal their non-binary identity, future work should approach the question of gender in a more nuanced manner and assess the relationship between gender and publication patterns beyond the women-men binary distinction. Moreover, these individual characteristics overlap and intersect with one another, thus creating specific challenges for those who represent more than one disadvantaged social category (e.g., Livingston et al., 2012). Future analyses exploring the patterns of (in)visibility of academic work would do well to take into account the intersectionality perspective (Crenshaw, 1989).

### Practical implications

Gender inclusiveness in special issues of leading journals can be achieved through introducing journal policies that bring potential bias into the awareness of the special issue editorial teams. First, journal editors can encourage mixed-gender guest editorial teams. Second, editors of special issues could be asked to actively encourage a broad range of authors to submit their work in order to minimize the bias through similarity. Finally, similar to minimizing gender bias in hiring by broadening the scope of shortlisted candidates (Lucas et al., 2021), introducing journal policies aimed at broadening the scope of articles in the first round (that is, those that are sent out to the reviewers) could potentially be helpful in mitigating gender bias by design. In contrast to editors, for reviewers, the names and, thus, the gender of the authors could be blinded, thereby making it less likely for reviewers to reveal a bias by design and approve male-authored works to a higher extent than those that are female-authored.

### Closing remarks

When discussing gender bias in science, factors that are typically brought up to explain the different career trajectories of women and men include gender bias outside academia—that is, a necessity for female academics to be engaged in societally expected duties, such as child-rearing and gender stereotypes that hinder women’s progress, as they are seen as less fit to the academic roles than men (Gruber et al., 2021). Recent research has indicated that, in addition to gender biases operating inside and outside of academia, there can also be intergroup processes at play that contribute to representation bias (Hofstra et al., 2020; Johnson et al., 2017). Within the field of psychology, however, this rarely gets labeled as male ingroup bias, making this reason for gender discrimination in academia an elephant in the room. The difficulty in talking about it seems to reflect a broader tendency of the new feminism to be considerate when it comes to “the man question.” Due to the feminist values of equality and inclusiveness, a moral obligation has been raised for the movement to *bridge the gender gap* and include men (Schacht et al., 2004). In the same vein, but more practically, it has been argued that, without men, feminism will simply not succeed (Hebert, 2007). This leads to a certain paradox: addressing male ingroup bias can be seen as antagonizing, whereas not addressing it leaves gender equality progressing at a slower pace, for instance, by not raising awareness of the issue.

This is particularly important, as gender disparity in many cases is not an instance of ill will. The preference for male scholars among men may not be an apparent hostile bias toward women but rather an incarnation of benevolent sexism and cultural notions regarding women’s fit to academia and perhaps also the unconscious wish for things to stay as they are (i.e., particularly welcoming for the ingroup). Regardless of the intentions behind this bias, it can seriously affect women’s career trajectories. Making men aware of this bias in academia can potentially limit it and speed up gender equality processes. Therefore, it deserves the attention of the academic community and calls for taking measures to mitigate it, such as promoting gender diversity in editorial teams. By introducing policies that bring awareness to and minimize potential gender bias, social psychology journals could model inclusiveness standards in the social sciences, thus contributing to changing standards within and beyond academia.
